# Evidence from paranoid schizophrenia for more than one component of theory of mind

**DOI:** 10.3389/fpsyg.2015.01643

**Published:** 2015-10-30

**Authors:** Peter Scherzer, André Achim, Edith Léveillé, Emilie Boisseau, Emmanuel Stip

**Affiliations:** ^1^Département de Psychologie, Université du Québec à MontréalMontréal, QC, Canada; ^2^Institut des sciences cognitives, Université du Québec à MontréalMontréal, QC, Canada; ^3^Hôpital du Sacré-Coeur de Montréal et sa FondationMontréal, QC, Canada; ^4^Hôpital Rivière-des-PrairiesMontréal, QC, Canada; ^5^Centre Hospitalier de l’Université de MontréalMontréal, QC, Canada

**Keywords:** schizophrenia, paranoid symptoms, theory of mind, executive functions

## Abstract

We previously reported finding that performance was impaired on four out of five theory of mind (ToM) tests in a group of 21 individuals diagnosed with paranoid schizophrenia (pScz), relative to a non-clinical group of 29 individuals (Scherzer et al., 2012). Only the Reading the Mind in the Eyes Test did not distinguish between groups. A principal components analysis revealed that the results on the ToM battery could be explained by one general ToM factor with the possibility of a latent second factor. As well, the tests were not equally sensitive to the pathology. There was also overmentalization in some ToM tests and under-mentalisation in others. These results led us to postulate that there is more than one component to ToM. We hypothesized that correlations between the different EF measures and ToM tests would differ sufficiently within and between groups to support this hypothesis. We considered the relationship between the performance on eight EF tests and five ToM tests in the same diagnosed and non-clinical individuals as in the first study. The ToM tests shared few EF correlates and each had its own best EF predictor. These findings support the hypothesis of multiple ToM components.

## Introduction

### Theory of Mind

Theory of mind (ToM) is but one component of social cognition Green et al. (2008). ToM is defined as the ability to attribute, correctly or incorrectly, beliefs, knowledge, feelings or intentions to others, in order to understand and predict their behavior (Perner, 1991; Perner and Lang, 2000; Green et al., 2008). The discovery of this ability coupled with the large inventory of tests used as a measure, allowed researchers to make strides in the understanding of atypical development and specifically in schizophrenia (Scz) (Frith, 1992; Frith and Corcoran, 1996; Langdon and Coltheart, 1999; Champagne et al., 2005; Champagne-Lavau et al., 2006, 2007; Uhlhaas et al., 2006; Martino et al., 2007).

### Theory of Mind and Schizophrenia

Research in social cognition in Scz has revealed reliable and large impairments in understanding first and second order false beliefs (Bora et al., 2009a; Bozikas et al., 2011), understanding indirect messages (Corcoran et al., 1995; Greig et al., 2004), inferring affect based on photos of the area around the eyes (Baron-Cohen et al., 2001) identifying irony and faux pas (Shamay-Tsoory et al., 2005; Chung et al., 2014), and making inferences concerning real time social interactions (Bazin et al., 2009; Ouellet et al., 2010; Montag et al., 2011).

### Executive Functions and ToM in Schizophrenia

Executive functions are considered to be a critical cognitive mediator for ToM (Perner and Lang, 2000). The link between executive deficits and ToM impairment may be explained by difficulty in inhibiting one’s own perspective and distinguishing it from others (Ruby and Decety, 2003). A difficulty in making non-literal interpretations may be due to a difficulty in inhibiting a usual interpretation (Leslie et al., 2004), a lack of flexibility that is reflected in difficulties judging the relative importance of each aspect of a script and attributing the appropriate importance to the pertinent information (Channon and Crawford, 2000).

Significant correlations have been found between a large variety of EF tests and ToM in patients with Scz (see for example Langdon et al., 2001; Bell and Mishara, 2006; Bora et al., 2006a; but see Lysaker et al., 2008 for a contrary opinion and results). Pickup (2008) analyzed 17 studies, eight of which reported a significant correlation between ToM and EF. Although there is a link between the two, he found that the patients were impaired on tests of ToM compared with control subjects, even when EF were factored out. On the other hand, he found that EF shared 65% of the variance on ToM tests in the clinical groups while there was no significant correlation in the non-clinical group (Pickup, 2008).

Bora et al. (2006a) used a battery to probe the relationship between insight into illness, ToM (RMET, first and second order false belief stories) and EF (WCST, Digit Span, verbal fluency, letter-number sequencing). RMET correlated with Digit Span backward and letter-number sequencing, but not WCST. Second order false beliefs correlated with letter-number sequencing, WCST perseveration and categories, but not with Digit Span backward. These results could lead one to consider that there are likely different ToM components as different ToM tests load differentially on EF tests. These discrepancies in results and divergent approaches in the analyses point to inconsistencies in the literature and raise questions about the structure or content of what is being measured.

Montag et al. (2011) identified one ToM component the cognitive/emotional content (see also Shamay-Tsoory et al., 2007). Hynes et al. (2006) identified a differential activation pattern depending on whether the social perspective-taking task was emotional or cognitive, with activation of the medial orbitofrontal lobe distinguishing between the two conditions. Shamay-Tsoory et al. (2006) found that patients with ventromedial prefrontal lesions performed better in the cognitive than in the emotional condition. The differentiation between the two components was further confirmed using ToM tests (Shamay-Tsoory et al., 2006, 2007; See Abu-Akel and Shamay-Tsoory, 2011 for a résumé of the neural circuitry of the components that they identify that include cognitive and emotional components). Salvatore et al. (2008, p. 193, paragraph 1) present an argument, based on data from different populations, in support of a multi- component ToM. However, the clinical evidence extracted from an interview with two patients diagnosed with schizophrenia leaves the debate unresolved.

### Objectives and Hypotheses

The present study is an attempt to reexamine the link between ToM (faux pas, lies, indirect messages, inferring facial expressions of emotions etc) and EF (cognitive flexibility, deductive reasoning, etc), in a group of patients diagnosed with paranoid schizophrenia (pScz) to determine if different EF measures, are equally good predictors of performance on a battery of ToM tests and if the ToM tests share the same relationship with the EF measures. We predict that although the clinical group will be impaired relative to a non-clinical group on the ToM measures, with the exception of the RMET (Scherzer et al., 2012), that performance on some ToM measures better distinguish between the two groups than others.

We further predict that performance on all the EF tests will be impaired in the pScz group relative to the non-clinical group but the pScz group will perform better on some EF measures than others. Finally, we predict that correlations between the different EF measures and ToM tests, will differ sufficiently within and between groups to support the contention that there is more than one component to ToM.

## Materials and Methods

The ethics committee of the Département de psychologie, Université du Québec à Montréal and the ethics and scientific committee (Comité d’éthique de la recherché) of the Hôpital Louis H. Lafontaine (recently renamed Institut Universitaire en Santé Mentale de Montréal) approved the protocol. Informed written consent was obtained from each subject prior to study entry.

### Participants

Twenty-one patients diagnosed with pScz and a group of 29 non-clinical individuals were recruited for the study (more details in Scherzer et al., 2012). The subjects were all males, between 18 and 35 years old, either native French speakers or having received all of their schooling in French. Their IQ (VIQ and PIQ, FSIQ) was ≥85. Individuals with Axis I and/or II comorbidity, neurologic problems, head trauma, alcoholism, substance abuse, or dependence, non-corrected visual deficits as determined by the medical records, were excluded from the study. As well, the attending psychiatrists verified that the participants were not under the influence of any recreational drugs or alcohol prior to experimentation.

The diagnosis of pScz was made by the attending psychiatrists and confirmed by ES according to DSM-IV (American Psychiatric Association [APA], 1994) diagnostic criteria. The clinical group was recruited from the outpatient clinic for young adults with psychosis of the Institut Universitaire en Santé Mentale de Montréal. Their medication had to be stable 2 weeks prior to data collection.

The non-clinical group was recruited from the community via posters, word-of-mouth and from talking to groups of individuals. Their socio-demographic profile (age, education, parental education) was comparable to that of the clinical group and their immediate family history (parents, siblings) had to be free of schizophrenia and other psychosis related disorders. They had to be free of Axis I and II comorbidity, neurological problems, head trauma, alcoholism, substance abuse, or dependence, non-corrected visual deficits. This information was obtained during an extensive telephone interview with the potential candidates.

### Measures

#### Clinical Evaluation (Kay et al., 1987)

The clinical group was evaluated using the *Positive and Negative Syndrome Scale* (PANSS) during a semi-structured interview. A second psychiatrist independently validated the ratings on the PANSS. The rated >4 on one or more of the following characteristic symptoms on the PANSS: grandiose ideas, delusions, or hallucinations, and persecutory ideation.

#### Neuropsychological Measures

##### WAIS-III (Wechsler, 1997)

An abridged French version of the WAIS-III (Pilgrim et al., 1999) was used to evaluate the intelligence of the participants in order to control for any potential contribution of this variable to the ToM measures. An estimate of VIQ was obtained using the following subtests: Information, Similarities, Digit Span, and Arithmetic. An estimate of PIQ was obtained using the following subtests: Picture Completion, Block Design Substitution.

##### Theory of mind tasks

Theory of mind was measured using five different tests: reading the mind in the eyes test (RMET; Baron-Cohen et al., 2001), Hinting Task (Corcoran et al., 1995; Marjoram et al., 2005), Strange Stories (Happé et al., 1998), and Faux pas (Stone et al., 1998) and Conversations and Insinuations (C and I; Ouellet et al., 2010).

###### Reading the mind in the eyes test (Baron-Cohen et al., 2001)

The RMET is a first order false belief test of recognition of mental states and emotions (Baron-Cohen et al., 2001; Craig et al., 2004). It consists of 36 images of the facial area around the eyes, each image illustrating a different mental state. The test has been found to distinguish between patients with schizophrenia and non-clinical participants (Bora et al., 2008; Kettle et al., 2008). The French version of the multiple choices was taken from the web site www.autismresearchcenter.com.

###### Hinting task (Corcoran et al., 1995; Marjoram et al., 2005)

The Hinting Task is a verbal measure of first and second order false beliefs (Bora et al., 2009a,b). It tests the ability to infer the real intentions behind indirect messages of the speaker (Corcoran et al., 1995; Bliksted et al., 2014). There are two versions of this test, each having 10 stories of social interactions in which one person sends an indirect message to another. These two versions were found to distinguish between patients with schizophrenia (Uhlhaas et al., 2006) with a high level of social functioning and those with a low level of social functioning (Bora et al., 2006b), between patients with schizophrenia and paranoid symptoms and patients with negative symptoms (Bora et al., 2008). A combined score derived from both versions were used in this study.

###### Strange stories (Happé, 1994; Happé et al., 1998)

This test consists of eight stories requiring an inference concerning the mental state of a protagonist (Happé et al., 1998) and eight control stories requiring a physical inference. This test was found to distinguish between subjects at high risk for schizophrenia and non-clinical subjects (Chung et al., 2008), as well as between patients with schizophrenia and non-clinical subjects (Langdon and Ward, 2009, 2010).

###### Faux Pas (Stone et al., 1998; Baron-Cohen et al., 1999)

This test consists of 10 stories describing the interaction between two people, one of whom unknowingly makes a comment that is insulting or hurtful, about the other person. It has been found to distinguish between schizophrenic patients with and without a history of violence (Abu-Akel and Abushua’leh, 2004) as well as between schizophrenic patients with negative symptoms and non-clinical control participants (Martino et al., 2007).

###### Conversations and insinuations (C and I; Ouellet et al., 2010)

This test is composed of four self-contained clips of approximately 2 min duration each, taken from popular French TV programs (see Ouellet et al., 2010 for details). The subject is required to make inferences in order to understand the social interactions, indirect messages, faux pas, white lies, and sarcasm used in the conversation. Each scene is independent of the others and does not require any further information in order to understand the content, nor having viewed previous episodes of the program. See Ouellet et al. (2010) for a more complete description of C and I.

##### Executive Function Battery

A battery of EF was used to assess speed of processing (Reitan and Wolfson, 1985), cognitive flexibility (Trails B–A, see Lezak et al., 2012) and inhibition (Hayling Sentence Completion Test, Burgess and Shallice, 1997); (Wisconsin Card Sorting Test – Abridged, Kongs, 2000; D-KEFS Stroop, Delis et al., 2001), verbal fluency (D-KEFS Letter and Category Fluency. Delis et al., 2001), Hayling Sentence Completion Test (Burgess and Shallice, 1997), deductive reasoning (Brixton Spatial Anticipation Task, Burgess and Shallice, 1997), problem solving (Tower of London, see Culberston and Zillmer, 2001) and planning (BADS Zoo Map, Wilson et al., 1996).

### Procedure

The entire battery required two sessions of approximately 2 h each to administer. The sequence of tests was counterbalanced between subjects. At the end of the testing, each participant received eight dollars in compensation. Participants in the clinical group were tested in the hospital whereas those in the non-clinical group were tested in a university laboratory.

### Statistical Analyses

Analyses of covariance (ANCOVA) adjusted for FSIQ and effect size ($\eta_{p}^{2}$) were used to compare the performances between groups on each ToM test. Student *t*-tests or analyses of covariance (ANCOVA) adjusted for IQ and education where appropriate, were used to compare the performance of the two groups on the measures derived from the tests of executive functions. Partial Pearson correlations within groups were used to examine the shared variance between EF and ToM measures, after controlling for FSIQ if pertinent.

## Results

### Statistical Analyses

All the transformations used are noted in detail in the subtext of the relevant tables, where appropriate.

### Between Group Comparisons of Socio-demographic Characteristics

Student *t*-tests revealed significant group differences for age, education and IQ measures (**Table 1**).

**Table 1:** Group comparisons between paranoid schizophrenic (pScz) and non-clinical groups.

	pScz	Non-clinical	
		
	*n* = 21	*n* = 29	*t*(48)
	*M* (*SD*)	*M* (*SD*)	
Age (yr)	25.71 (4.44)	23.07 (3.2)	2.45^∗^
Education, subjects (yrs)	10.71 (1.55)	12.03 (1.18)	-3.42^a∗∗^
Education, parents (yrs)	11.94 (2.06)	12.91 (2.45)	-1.38ns
FSIQ	99.95 (11.58)	110 (11.04)	-3.11^b∗∗^
VIQ	100.81 (11.84)	109.07 (14.82)	-2.47^c∗^
PIQ	98.24 (12.09)	111.03 (13.83)	-3.40^∗∗^
Age at diagnosis (yrs)	21.57 (2.50)		
Chronicity (yrs)	5.67 (5.13)		
PANSS positive symptoms (0–49)	21.19 (2.46)		
Hallucinations/delusions (0–7)	4 (1.34)		
Grandiose ideas (0–7)	2,38 (0,74)		
Persecutory ideation (0–7)	3,86 (1,15)		
PANSS negative symptoms (0–49)	17,48 (4,72)		
PANSS psychopathology (0–112)	39,43 (6,67)		

*^∗^*p* < 0.05; ^∗∗^*p* < 0.01*.

*^a^Given positive skewness of age in patients (*z* = 1.059/0.501) and negative (*z* =−1.333/0.434 =−3.07) in controls, the test was confirmed by Mann–Whitney *U* = 134.5, *p* = 0.001*.

*^b^FSIQ was positively skewed in controls (*z* = 1.068/0.434 = 2.46) and acceptably symmetrical in patients (*z* = 0.837/0.501 = 1.67). The former is attributed to sampling bias rather that to scale non-linearity. Group difference is confirmed by Mann–Whitney *U* = 153, *p* = 0.003*.

*^c^Verbal IQ is positively skewed in patients (*z* = 1.085/0.501 = 2.16) and acceptably symmetrical in controls (*z* = 0.242/0.434). The group difference is still significant by Mann–Whitney *U* = 190, *p* = 0.024*.

### Comparison between Groups on ToM Tests

Given the group differences in background variables that could influence the ToM or EF variables, the group differences on these variables were tested first by including education and FSIQ as covariables. Only covariables declared significant predictors of the dependent ToM variable at *p* < 0.05 were kept and the homogeneity of regression slopes was verified. If homogeneity was rejected (i.e., covariable × dependent variable interaction significant at *p* < 0.05), the covariable was excluded and the situation flagged in the tabled report. The standard deviations reported are the original ones, not reduced by the retained covariables, if any.

Two ToM variables were significantly negatively skewed in controls because of ceiling effects. The skewness index was at *z* = -1.486/0.434 = -3.42 for Strange Stories, with 18 of the 29 controls at ceiling. For Faux Pas, skewness was at *z* = -2.958/0.434 = -6.82, with 11 at ceiling. For patients, skewness was, respectively, at *z* = -0.713/0.501 = -1.42 and *z* = -0.795/0.501 = -1.59. Without the participants performing at ceiling, the skewness for Strange Stories reduced to *z* = -0.294/0.661, but for Faux Pas it reduced only to *z* = -2.438/0.536 = -4.55. The latter value, in association with the negative skewness in the patients, warranted a scale transformation for this variable. The transformation L Faux Pas = 2-LG10(60.3-Faux Pas) brought skewness to *z* = 0.151/0.501 for the patients and to *z* = -0.282/0.434 all 29 controls and *z* = 0–698/0.536 for the 18 controls not at ceiling. The transformed version is used in the statistical analyses, and its group means are reported back transformed in the original scale.

The scores on the ToM tests were scaled for a maximum of 100 to allow for comparisons between tests prior to analyses. The effect size (rp^2^) was also calculated in order to identify those tests that best distinguished between the two groups. Hinting Task appear to be the most sensitive followed by C and I, Strange Stores, Faux Pas, and RMET, in that order. Only RMET did not distinguish between the groups (**Table 2**) and was not considered for further analysis.

**Table 2:** Group comparison on ToM measures scaled for maximum of 100 (adjusted for IQ and education if justified).

ToM tests	pScz (*n* = 21) *M* (*SD*)	Non-clinical (*n* = 29) *M* (*SD*)	Df	*F*	$\eta_{p}^{2}$(%)
Hinting task	72.3 (10.8)	92.6 (4.0)	1,48	87.66	64.6
C and I	52.3 (14.3)	73.8 (10,5)	1,47	38.76	45.2
Strange stories	64.6 (21.9)	94.6 (8.6)	1,48	44.97	48.4
Faux Pas	67.8 (22.8)	93.2 (10.5)	1,48	48.24	50.1
RMET	56.2 (8.0)	60.2 (11.0)	1,48	1.18	2.4

^a^*FSIQ would have been a significant covariate (*p* = 0.009) but is not included because homogeneity of slope was subsequently rejected (*p* = 0.008)*.

^b^*FSIQ covariable: *F*(1,47) = 12.04, *p* = 0.001*.

^c^*FSIQ would have been a significant covariate (*p* = 0.010) but is not included because homogeneity of slope was subsequently rejected (*p* = 0.006)*.

^d^*The back transformed means are 73.7 and 98.1*.

** p < 0.01.

### Group Comparisons of Executive Measures

Student *t*-tests or ANCOVA controlling for IQ and education, were used to compare the performance of the two groups on the 24 measures derived from the eight EF tests. Only the significant results are presented in **Table 3**.

**Table 3:** Comparisons between paranoid schizophrenic (pScz) and non-clinical groups’ executive function scores adjusted for IQ and education if justified.

Test	pScz (*n*-21) *M (SD)*	Non-clinical (*n* = 29) *M* (SD)	Df	*F* Covar	*F* Group	*$\eta_{p}^{2}$*
Digit Span^Q^ (DS) [Scaled Score (SS) forward and backward]	9.67 (2.13)	9.00 (2.82)	47	^Q^9.04	0.83	1.7
DS forward (SS)	6.72 (1.03)	6,24 (1.24)	47	^Q^5.57	1.92	3.9
DS backward (SS)	4,76 (1,29)	4.76 (1,55)	47	^Q^7.59	0.00	0.0
Similarities (SS)^a^	10.88 (2.33)	10.19 (3.61	48		2.72	5.4
Trails B–A (sec)^b^	41.43 (18.23)	34.07 (18.07)	47		2.18	4.4
Stroop interference (sec)^c^	50.73 (10.11)	50.86 (11.11)	47		1.45	3.0
Stroop interference (# errors)^d^	2.10 (3.03)	1.35 (2.37)	47		2.56	5.2
Stroop flexibility (sec)	59.00 (11.32)	59.62 (16.90)	46	^E^5.75	0.36	0.1
Stroop flexibility (# errors)^e^	2.07 (2.62)	2.82 (2.63)	46		0,89	0.28
Fluidity (phonological – # words)^e^	33.70 (11.08)	34.55 (11.02)	47		0.70	0.1
Fluidity (semantic – #words)^f^	36.02 (9.12)	39.41 (7.80)	45	^Q^7.70	1.58	3.4
Hayling control condition (sec)^g^	42.31 (18.73)	38.85 (18.81)	41		0.34	0.8
Hayling inhibition (# errors)^h^	8.06 (2.39)	4.27 (3.17)	47		29.83^∗∗^	38.8
Brixton aban^i^	2.5 (1.86)	1.79 (1.66)	42		1.68	3.8
Tower of London (TOL) (execution time: sec)^j^	187.95 (79.51)	192.5 (91.53)	45	^Q^5.84	0.83	1.8
TOL (# errors)^k^	0.40 (0.75)	0.21 (0.42)	46	n.a.		
TOL (planning time: sec)^l^	18.10 (19.96)	50.89 (32.58)	45.1	n.a.	*t* = 4.31^∗∗^	*d* = 1.01
TOL (moves)	38.35 (13.65)	16.68 (11.56)	46		35.26^∗∗^	43.4
Zoo map planning time (sec)^m^	30.05 (44.63)	23.79 (26.90)	46		0.28	0.6
Zoo Map (# errors)	1.05 (0.85)	0.55 (0.63)	46		5.49^∗^	10.7
Zoo map (execution time: sec)^n^	111.74 (62.76)	40.59 (33.15)	45	5.70	14.43^∗∗^	24.3
WCST (# categories)^o^	3.42 (1.54)	4.52 (0.92)	42		8.99^∗∗^	17.6
WCST (perseveration errors)^p^	7.95 (4.60)	4.96 (4.04)	42		7.06^∗^	14.4
WCST (non-perseveration errors NP)	6.84 (5.29)	4.90 (3.05)	42		4.63^∗^	9.9

^Q^*Mean adjusted for FSIQ*.

^E^*Mean adjusted for years of education*.

*^∗^*p* < 0.05, ^∗∗^*p* < 0.01*.

^a^*FSIQ covariable excluded for lack of homogeneity of regression slopes (*p* = 0.04)*.

*^b^One control missing. Score square root transformed, no constant added, to reduce skewness from 0.941 (*z* = 2.13) to 0.356 in controls (0.211 to -0.046 in patients). Back transformed means are 36.35 and 34.00*.

^c^*One patient missing. Because of excess skewness in controls (*z* = 1.323/0.434 = 3.05), transforming by SQRT(score-35) made the skewness within on standard error in both groups. Back transformed means are 52.47 and 48.10*.

^d^*One patient missing. Because of excess skewness in controls (*z* = 1.259/0.434 = 2.90), transforming by SQRT(score) made the skewness within on standard error in both groups. Back transformed means are 2.48 and 1.27*.

^e^*One patient missing*.

^f^*One subject missing in each group*.

^g^*Only available for 16 patients and 27 controls*.

^h^*One patient excluded as an outlier, being at *z* = -4.7 from the remaining of that group*.

^i^*Available for 16 patients and 28 controls. Because of excess skewness in controls (*z* = 1.406/0.441 = 3.19), transforming by SQRT(score+0.25) made the skewness within on standard error in each groups. Back transformed means are 2.65 and 1.96*.

^j^*Avaiable for 20 patients and 28 controls. This variable is positively skewed in both groups: *z* = 1.572/0.512 = 3.07 for the patients and *z* = 2.015/0.441 = 4.57 for the controls. The minimum observed was 96. The transformation LtolExec = LG10(score-80) brought the skewness index to.222 and -0.249, respectively. Tabled raw means do not take the FSIQ covariate into account. Back transformed covaried means are 156.38 and 173.33*.

^k^*Errors (0, 1, 2) are distributed 15, 2, 3 for the patients and 22, 6, 0 for the controls. With only two different observed values for the controls, no transformation can change the skewness. Because of this poor distribution, analysis of covariance is not applicable (n.a.). A chi-square test indicates that the two distributions do not differ significantly: χ^2^(2) = 5.13, *p* = .077*.

^l^*Tabled means are for 20 patients and 28 controls. But 12 of the patients had the minimum observed score of 10. Since the group variances differ, a *t*-test for unequal variances is reported. One patient was clearly an outlier for his group, with a score of 97 (quite normal for controls); without this patient, the mean was 13.95 with an SD of 7.5, which makes the outlier some 11 SD. above the mean of the remaining patients. Without him, group variance differ even more, the group difference gives *t*(31.1) = 5.78 and effect size, based on the SD of the controls, is *d* = 1.13. But with 12 of the remaining 19 patients at the observed minimum, all statistical tests based on normal distributions are of doubtful validity. In the 28 controls, skewness was good at *z* = 0.180/0.441*.

^m^*Zoo test not available for two patients. Planning time skewness is not acceptable in the patients (*z* = 1.678/0.524 = 3.20, with *z* = 0.814/0.434 = 1.88 in controls). There are several scores of 0 (10 of the 19 patients and 11 of the 29 controls). The transformation lzooplan = LG10(score+0.5) brings skewness to 0.229 and -0.283, respectively. Back transformed means are 4.01 and 5.91*.

^n^*Skewness is acceptable in patients (*z* = 0.118/0.524) but not in controls (*z* = 1.025/0.434 = 2.36). The observed minimum is 9. The transformation szooexec = SQRT(score-9) brings skewness to -0.343 and 0.304. Back transformed means are 89.10 and 35.32*.

^o^*Available for 19 patients and 25 controls. Skewness is negative in both groups and significantly different from zero in controls (*z* = -0.907/0.524 = -1.73 et *z* = -1.292/0.464 = -2.78). Scores vary from 0 to 6. Transforming into swctscat = 2-SQRT(6-score) preserves the original polarity without producing negative scores and brings skewness to -0.512 and 0.255, respectively. Back transformed means are 3.63 and 4.67*.

^p^*Skewness would be acceptable in patients (*z* = 0.665/0.524 = 1.27) but is not in controls (*z* = 1.665/0.464 = 3.59). Range is 1 to 17. Transforming into lwcEP = LG10(score+2) brought skewness to 0.227 and -0.042, respectively. Back transformed means are 7.02 and 4.50*.

^q^*Skewness was at *z* = 2.941/0.524 = 5.61 in patients and at *z* = 1.8/0.464 = 3.88 in controls. Range was 1 to 26. The transformation lwcstenp = LG10(score-0.8) brought skewness to 0.540 and -0.396, respectively. Back transformed means are 5.56 and 3.50*.

Performance of the clinical group was impaired compared with the non-clinical group, on 8 out of 24 measures derived from the eight EF tests. The effect size was largest for TOL planning time (tested by *t* for unequal variances, effect size using pooled variance *d* = 1.01 and using control variance *d* = 1.13) and TOL number of moves ($\eta_{p}^{2}$ = 43.4%) followed by Hayling inhibition errors ($\eta_{p}^{2}$ = 38.8%) and by Zoo Map execution time ($\eta_{p}^{2}$ = 24.3%).

### Relation between Performance on EF and ToM Measures

Correlations between ToM variables and EF variables were examined with Education and FSIQ as covariables where appropriate, first in patients and then in controls. Transformed versions were used, when indicated (see **Tables 2** and **3**). Only EF variables that have at least one significant correlation with a ToM variable are reported in **Table 4**.

**Table 4:** Correlations within and between pScz and non-clinical groups, between EF and ToM measures, with FSIQ partialled out (^a,b^) when it correlated significantly with that particular variable.

Group	EF variable	ToM variables			
		Hinting Task^a^	Strange Stories^a^	Faux Pas	C and I^ab^
PScz	Digit Span backward^a^	0.543^∗^	0.270	0.340	0.364
	Stroop flexibility time	-0.222	-0.617^∗∗^	-0.220	-0.546^∗^
	Hayling errors	-0.035	-0.239	0.167	-0.519^∗^
	Brixton aban	-0.452	-0.086	-0.369	-0.610^∗^
	Tower of London errors	-0.010	-0.629^∗∗^	-0.020	0.327
	Zoo map planning time	-0.035	0.141	-0.583^∗∗^	-0.186
Controls	Digit Span SS	-0.194	-0.096	0.076	0.441^∗^
	Digit Span forward	-0.007	-0.115	0.169	0.499^∗∗^
	Similarities^b^	0.033	0.041	0.411^∗^	0.230
	Trails B–A	-0.006	0.001	0.106	0.062
	Tower of London exec. time	-0.397^∗^	0.085	-0.161	-0.199

^∗^*p < 0.05, ^∗∗^p < 0.01*.

^a^*Correlated with FSIQ in patients*.

^b^*Correlated with FSIQ in controls*.

**Table 5** presents the percentage of variance *(*rp^2^*)* of each ToM measure that is explained by the scores on the respective EF tests.

**Table 5:** Best executive function predictors of performance on ToM tests.

ToM test	pScz group	rp^2^ (%)	Non-clinical group	rp^2^ (%)
Hinting Task	Digit span backward	29.6	TOL time	15.8
Strange Stories	TOL errors	39.6	-	
Faux Pas	Zoo Map planning time	33.98	Similarities (WAIS)	16.3
C and I	Brixton aban.	37.2	Digit Span	24.9

*TOL, Tower of London*.

There is no overlap between the best EF and ToM measures within groups. The shared variance differs between tests and groups, ranging from 29.6 to 44.6% in the clinical group and from 15.8 to 24.9% in the non-clinical group, which leaves 75.1 to 84.2% of the variance unaccounted for.

## Discussion

We first predicted that some ToM measures would be more sensitive and better discriminate between the pScz and non-clinical group and this hypothesis was confirmed. Hinting Task is the most sensitive [$\eta_{p}^{2}$ (%) = 64.6] while RMET does not distinguish between the two groups. Hinting Task measures indirect speech acts, requiring distinguishing between explicit, literal, unambiguous content and the intended, implicit, ambiguous content (Lukas, 2011; Bliksted et al., 2014). It requires a sharing of information, some knowledge of the conventions of conversation, the ability to infer the non-literal primary directive component of speech, i.e., the ability to identify and decode the attempt by the speaker to get the listener to do something (Searle, 1975; Hagoort and Indefrey, 2014). The complexity of the task or any of these requirements may explain the sensitivity of this measure. The other tests, C and I, Strange Stories, and Faux Pas are progressively less sensitive in distinguishing between the pScz and non-clinical groups.

In contrast to our prediction but in agreement with Chung et al. (2008), few of the measures used in the present study were sensitive to the pathology even after controlling for IQ. Of the 24 measures derived from the eight EF tests, only eight are significant. The most sensitive of these measures was Tower of London planning time. The clinical group took less time to plan their moves and as a consequence made more moves than the non-clinical group before finding a solution to the problem, although they made a comparable number of errors. However, when they took as much time as the non-clinical group to plan their moves on another task (Zoo Map) [$\eta_{p}^{2}$ (%) = 0.6], it took them significantly longer to find the solution [$\eta_{p}^{2}$ (%) = 24.3] and they made significantly more errors than the non-clinical group [$\eta_{p}^{2}$ (%) = 10.7].

Finally, we predicted that correlations between the different EF measures and ToM tests, would differ sufficiently within and between groups to support the contention that there is more than one component to ToM. As predicted each ToM test had its own best EF correlate. These findings as well as the differences between best EF predictor of ToM in the pScz and non-clinical groups would tend to support the contention that there is more than one ToM component.

A secondary goal of the study of ToM in clinical populations should be to help elucidate the processes involved in the pathology. To this end, and based on the content of the tests, we can derive the following composite image. Patients with paranoid schizophrenia have problems with on-line planning and anticipation of the consequences of their actions (TOL errors) and it is harder for them to switch from one mode of responding to another (Stroop Condition 4 – flexibility time; see also Ibáñez et al., 2014). They especially take more time to plan when confronted with a complex task that requires a lot of thought and planning before initiating any action (Tower of London planning time). They also are more likely to abandon any effort to deduce a rule when it changes without notice (Brixton).

At a social cognitive level, they have difficulties (1) correctly interpreting another’s state of mind in order to best be able to explain what one might consider unusual behavior in the context; (2) correctly perceiving and interpreting indirect messages; (3) detecting and understanding an inadvertent, inappropriate comment and the effect that this comment could provoke. These difficulties overlap well with the list of ToM abilities described by Lysaker et al. (2008).

If one is inclined to agree that there is more than one component to ToM then what is needed is a model of these components rather than a list. Such a model should be based on the developmental trajectory of the components, the dissociation of neural pathways, and the link between components at different stages of development (Tager-Flusberg and Sullivan, 2000; e.g., are first order beliefs a prerequisite to the development of second order beliefs?). One such model could be as follows: first→second order beliefs ↔(*NB – indicating the possibility of an overlap.* See for example Weimer et al., 2012) emotional beliefs, → order beliefs of intention (see for example Baron-Cohen et al., 1999; Brüne et al., 2007; Martino et al., 2007; Zalla et al., 2009).

## Limitations

The results of this study provide evidence for a multicomponent model of ToM and a message to researchers for the need to identify what these components might be and how they may be affected in various clinical populations. However, there is an important need for replication studies given the relatively small sample and the fact that the results of this study, taken at face value, could be interpreted as being attributable to chance (24 comparisons between two groups, eight significant results – **Table 3**). However, it should be noted that seven of the eight measures were derived from just three out of the eight tests: Wisconsin Card Sorting Test (3), Zoo Map (2), Tower of London (2). If the distribution was random one would expect the results from more EF tests to be significant. Also, chance remains a viable alternative explanation for most of the correlations in **Table 4** (four ToM measures, 24 EF measures and two groups: 11 significant results, 4 of which were at the 0.01 level). These results remain to be confirmed by others.

Finally, the differences between the results on the EF measures and the correlations between the measures and ToM measures may be attributable to the limited psychometric qualities of these tests (see Green et al., 2008) and the fact that we did not control for anxiety (Lysaker et al., 2010; Achim et al., 2011, 2013). There is also a lack of important information concerning the psychometric qualities of each ToM test (Green et al., 2008) although concomitant validity in terms of predicting group membership appears to be acceptable, as does test–retest reliability. Finally, the number of EF and ToM measures used in this study while numerous, do not account for all tests and measures used in this domain. It is quite possible that the inclusion of other tests would modify the findings. More participants from this clinical group need to be tested on this and similar batteries in order to determine the reliability and validity of the results and the applicability of the clinical description to the population.

## Conflict of Interest Statement

The authors declare that the research was conducted in the absence of any commercial or financial relationships that could be construed as a potential conflict of interest.

## References

[B1] Abu-Akel A. Abushua’leh K. (2004). ‘Theory of mind’ in violent and nonviolent patients with paranoid schizophrenia. *Schizophr. Res.* 69 45–53. 10.1016/S0920-9964(03)00049-5 15145470

[B2] Abu-Akel A. Shamay-Tsoory S. (2011). Neuroanatomical and neurochemical bases of theory of mind. *Neuropsychologia* 49 2971–2984. 10.1016/j.neuropsychologia.2011.07.012 21803062

[B3] Achim A. M. Maziade M. Raymond É Olivier D. Mérette C. Roy M.-A. (2011). How prevalent are anxiety disorders in schizophrenia? A meta-analysis and critical review on a significant association. *Schizophr. Bull.* 37 811–821. 10.1093/schbul/sbp148 19959704 PMC3122284

[B4] Achim A. M. Ouellet R. Lavoiek M.-A. Vallières C. Jackson P. L. Roy M.-A. (2013). Impact of social anxiety on social cognition and function in patients with recent-onset schizophrenia spectrum disorders. *Schizophr. Res.* 145 75–81. 10.1016/j.schres.2013.01.012 23415473

[B5] American Psychiatric Association [APA] (1994). *Diagnostic and Statistical Manual of Mental Disorders*, Fourth Edn Washington, DC: American Psychiatric Association.

[B6] Baron-Cohen S. O’Riordan M. Stone V. Jones R. Plaisted K. (1999). A new test of social sensitivity: detection of faux pas in normal children and children with Asperger syndrome. *J. Autism. Dev. Disord.* 29 407–418. 10.1023/A:1023035012436 10587887

[B7] Baron-Cohen S. Wheelwright S. Hill J. Raste Y. Plumb I. (2001). The “reading the mind in the eyes” test revised version: a study with normal adults, and adults with Asperger syndrome or high-functioning autism. *J. Child Psychol. Psychiatry* 42 241–251. 10.1111/1469-7610.00715 11280420

[B8] Bazin N. Brunet-Gouet E. Bourdet C. Kayser N. Falissard B. Hardy-Baylé M.-C. (2009). Quantitative assessment of attribution of intentions to others in schizophrenia using an ecological video-based task: a comparison with manic and depressed patients. *Psychiatry Res.* 167 28–35. 10.1016/j.psychres.2007.12.010 19346006

[B9] Bell M. D. Mishara A. L. (2006). Does negative symptom change relate to neurocognitive change in schizophrenia? Implications for targeted treatments. *Schizophr. Res.* 81 17–27. 16297601 10.1016/j.schres.2005.09.016

[B10] Bliksted V. Fagerlund B. Weed E. Frith C. Videbech P. (2014). Social cognition and neurocognitive deficits in first-episode schizophrenia. *Schizophr. Res.* 153 9–17. 10.1016/j.schres.2014.01.010 24480016

[B11] Bora E. Eryavuz A. Kayan B. Sungu G. Veznedarogly B. (2006a). Social functioning, theory of mind and neurocognition in patients with schizophrenia: mental state decoding may be a better predictor of social functioning than mental state reasoning. *Psychiatry Res.* 145 95–103. 10.1016/j.psychres.2005.11.003 17074402

[B12] Bora E. Sehitoglu G. Aslier M. Atabay I. Veznedarogly B. (2006b). Theory of mind and unawareness of illness in schizophrenia: is poor insight a mentralising deficit. *Eur. Arch. Psychiatry Clin. Neurosci.* 257 104–111. 17171312 10.1007/s00406-006-0681-3

[B13] Bora E. Gökçen S. Kayahan B. Veznedaroglu B. (2008). Deficits of social-cognitive and social-perceptual aspects of theory of mind in remitted patients with schizophrenia: effect of residual symptoms. *J. Nervous Ment. Dis.* 196 95–99. 10.1097/NMD.0b013e318162a9e1 18277216

[B14] Bora E. Yucel M. Pantelis C. (2009a). Theory of mind impairment in schizophrenia: meta-analysis. *Schizophr. Res.* 109 1–9. 10.1016/j.schres.2008.12.020 19195844

[B15] Bora E. Yücel M. Pantelis C. (2009b). Theory of mind impairment: a distinct trait-marker for schizophrenia spectrum disorders and bipolar disorder? *Acta Psychiatr. Scand.* 120 253–264. 10.1111/j.1600-0447.2009.01414.x 19489747

[B16] Bozikas V. P. Giannakou M. Kosmidis M. H. Kargopoulos P. Kioseoglou G. Liolios D. (2011). Insights into theory of mind in schizophrenia: the impact of cognitive impairment. *Schizophr. Res.* 130 130–136. 10.1016/j.schres.2011.04.025 21602031

[B17] Brüne M. Abdel-Hamid M. Lehmkämper C. Sonntag C. (2007). Mental state attribution, neurocognitive functioning, and psychopathology: What predicts poor social competence in schizophrenia best? *Schizophr. Res.* 92 151–159. 10.1016/j.schres.2007.01.006 17346931

[B18] Burgess P. W. Shallice T. (1997). *The Hayling and Brixton tests.* London: Harcourt Assessment.

[B19] Champagne M. Stip E. Joanette Y. (2005). Cognitive determinants of pragmatic deficits in right brain damaged and schizophrenic individuals: a comparative study. *Brain Cogn.* 5:278.

[B20] Champagne-Lavau M. Stip E. Joanette Y. (2006). Social cognition deficit in schizophrenia: accounting for pragmatic deficits in communication abilities? *Curr. Psychiatry Rev.* 2 309–315. 10.1016/j.encep.2007.04.005

[B21] Champagne-Lavau M. Stip E. Joanette Y. (2007). Language functions in right-hemisphere damage and schizophrenia: apparently similar pragmatic deficits may hide profound differences. *Brain* 130:e67 10.1093/brain/awl311 17235120

[B22] Channon S. Crawford S. (2000). The effects of anterior lesions on performance on a story comprehension test: left anterior impairment on a theory of mind-type task. *Neuropsychologia* 38 1006–1017. 10.1016/S0028-3932(99)00154-2 10775711

[B23] Chung Y. S. Barch D. Strube M. (2014). A met-analysis of mentalizing impaairments iin adults with schizophrenia and autism spectrum disorder. *Schizophr. Bull.* 40 602–616. 10.1093/schbul/sbt048 23686020 PMC3984506

[B24] Chung Y. S. Kang D.-H. Shin N. Y. Yoo S. Y. Kwon J. S. (2008). Deficit of theory of mind in individuals at ultra-high-risk for schizophrenia. *Schizophr. Res.* 99 111–118. 10.1016/j.schres.2007.11.012 18096371

[B25] Corcoran R. Mercer G. Frith C. D. (1995). Schizophrenia symptomatology and social inference: investigating “theory of mind” in people with schizophrenia. *Schizophr. Res.* 17 5–13. 10.1016/0920-9964(95)00024-G 8541250

[B26] Craig J. S. Hatton C. Craig F. B. Bentall R. P. (2004). Persecutory beliefs, attributions and theory of mind: comparison of patients with paranoid delusions. Asperger’s syndrome and healthy controls. *Schizophr. Res.* 69 29–33. 15145468 10.1016/S0920-9964(03)00154-3

[B27] Culberston W. C. Zillmer E. (2001). *Tower of London.* Ontario: Toronto Multi-Health Systems.

[B28] Delis D. C. Kaplan E. Kramer J. H. (2001). *Delis-Kaplan Executive Function System.* San Antonio, TX: The Psychological Corporation.

[B29] Frith C. D. (1992). *The Cognitive Neuropsychology of Schizophrenia.* Hove: Lawrence Erbaum Associates.

[B30] Frith C. D. Corcoran R. (1996). Exploring ‘theory of mind’ in people with schizophrenia. *Psychol. Med.* 26 521–530. 10.1017/S0033291700035601 8733211

[B31] Green M. F. Penn D. L. Bentall R. Carpenter W. T. Gaebel W. Gur R. C. (2008). Social cognition in schizophrenia: a NIMH workshop on definitions, assessment, and research opportunities. *Schizophr. Bull.* 34 1211–1220. 10.1093/schbul/sbm145 18184635 PMC2632490

[B32] Greig T. C. Bryson G. J. Bell M. D. (2004). Theory of mind performance in schizophrenia: diagnostic, symptom, and neuropsychological correlates. *J. Nervous Ment. Dis.* 192 12–18. 10.1097/01.nmd.0000105995.67947.fc 14718771

[B33] Hagoort P. Indefrey P. (2014). The neurobiology of language beyond single words. *Annu. Rev. Neurosci.* 37 347–362. 24905595 10.1146/annurev-neuro-071013-013847

[B34] Happé F. G. E. (1994). An advanced test of theory of mind: understanding of story characters’ thoughts and feelings by able autistic, mentally handicapped, and normal children and adults. *J. Dev. Disord.* 24 129–154. 10.1007/BF02172093 8040158

[B35] Happé F. G. E. Winner E. Brownell H. (1998). The getting of wisdom: theory of mind in old age. *Dev. Psychol.* 34 358–362. 10.1037/0012-1649.34.2.358 9541787

[B36] Hynes C. A. Baird A. A. Grafton S. T. (2006). Differential role of the orbital frontal lobe in emotional versus cognitive perspective-taking. *Neuropsychologia* 44 374–383. 10.1016/j.neuropsychologia.2005.06.011 16112148

[B37] Ibáñez A. Aguado J. Baez S. Huepe D. Lopez V. Ortega R. (2014). From neural signatures of emotional modulation to social cognition: individual differences in healthy volunteers and psychiatric participants. *Soc. Cogn. Affect. Neurosci.* 9 939–950. 10.1093/scan/nst067 23685775 PMC4090956

[B38] Kay S. R. Fiszbein A. Opfer L. A. (1987). The positive and negative syndrome scale (PANSS) for schizophrenia. *Schizophr. Bull.* 13 261–276. 10.1093/schbul/13.2.261 3616518

[B39] Kettle J. W. L. O’Brien-Simpson L. Allen N. B. (2008). Impaired theory of mind in first-episode schizophrenia : comparison with community, university and depressed controls. *Schizophr. Res.* 99 96–102. 10.1016/j.schres.2007.11.011 18155447

[B40] Kongs S. K. (2000). *Wisconsin Card Sorting Test, 64 Cards Version.* Lutz, FL: Psychological Assessment Resources.

[B41] Langdon R. Coltheart M. (1999). Mentalising, schizotypy and schizophrenia. *Cognition* 71 43–71. 10.1016/S0010-0277(99)00018-9 10394709

[B42] Langdon R. Coltheart M. Ward P. B. Catts S. V. (2001). Mentalising, executive planning and disengagement in schizophrenia. *Cogn. Neuropsychiatr.* 6 81–108. 10.1080/13546800042000061

[B43] Langdon R. Ward P. B. (2009). Taking the perspective of the other contributes to awareness of illness in schizophrenia. *Schizophr. Bull.* 35 1003–1011. 10.1093/schbul/sbn039 18495647 PMC2728812

[B44] Langdon R. Ward P. B. (2010). Reasoning anomalies associated with delusions in schizophrenia. *Schizophr. Bull.* 36 321–330. 10.1093/schbul/sbn069 18622010 PMC2833109

[B45] Leslie A. M. Friedman O. German T. P. (2004). Core mechanisms in ‘theory of mind’. *Trends Cogn. Sci.* 8 528–533. 10.1016/j.tics.2004.10.001 15556021

[B46] Lezak M. Howieson D. B. Bigler E. D. Tranel D. (2012). *Neuropsychological Assessment*, 5th Edn New York: Oxford University Press.

[B47] Lukas K. (2011). Socializing messages in blue-collar families: communicative pathways to social mobility and reproduction. *Western J. Commun.* 75 95–121. 10.1080/10570314.2010.536964

[B48] Lysaker P. H. Salvatore G. Grant M. L. A. Procacci M. Olesek K. L. Buck K. D. (2010). Deficits in theory of m ind and social anxiety as independent paths to paranoid features in schizophrenia. *Schizophr. Res.* 124 81–85. 10.1016/j.schres.2010.06.019 20655179

[B49] Lysaker P. H. Warman D. M. Dimaggio G. Procacci M. LaRocco V. A. Clark L. K. (2008). Metacognition in schizophrenia: associations with multiple assessments of executive function. *J. Nerv. Ment. Dis.* 196 384–389. 10.1097/NMD.0b013e3181710916 18477880

[B50] Marjoram D. Gardner C. Burns J. Miller P. Lawrie S. M. Johnstone E. C. (2005). Symptomatology and social inference: a theory of mind study of schizophrenia and psychotic affective disorder. *Cogn. Neuropsychiatr.* 10 347–359. 10.1080/13546800444000092 16571466

[B51] Martino D. J. Bucay D. Burman J. T. Allegri R. F. (2007). Neuropsychological frontal impairments and negative symptoms in schizophrenia. *Psychiatry Res.* 152 121–128. 10.1016/j.psychres.2006.03.002 17507100

[B52] Montag C. Dziobek I. Richter I. S. Neuhaus K. Lehmann A. Sylla R. (2011). Different aspects of theory of mind in paranoid schizophrenia: evidence from a video-based assessment. *Psychiatry Res.* 186 203–209. 10.1016/j.psychres.2010.09.006 20947175

[B53] Ouellet J. Scherzer P. B. Rouleau I. Métras P. Bertrand-Gauvin C. Djerroud N. (2010). Assessment of social cognition in patients with multiple sclerosis. *J. Int. Neuropsychol. Soc.* 16 287–296. 10.1017/S1355617709991329 20167136

[B54] Perner J. (1991). *Understanding the Representational Mind.* Cambridge, MA: MIT Press.

[B55] Perner J. Lang B. (2000). “Theory of mind and executive function: is there a developmental relationship?,” in *Understanding Other Minds: Perspectives from Autism and Developmental Cognitive Neuroscience*, Chapt. 4 2nd Edn, eds Baron-Cohen S. Tager-Flusberg H. Cohen D. (Oxford: Oxford University Press).

[B56] Pickup G. J. (2008). Relationship between theory of mind and executive function in schizophrenia: a systematic review. *Psychopathology* 41 206–213. 10.1159/000125554 18408416

[B57] Pilgrim B. M. Meyers J. E. Bayless J. Whetstone M. M. (1999). Validity of the ward seven-subtest wais-III short form in a neuropsychological population. *Appl. Neuropsychol.* 6 243–246. 10.1207/s15324826an0604_7 10635439

[B58] Reitan R. M. Wolfson D. (1985). *The Halstead-Reitan Neuropsychological Test Battery.* Tucson, AZ: Neuropsychology Press.

[B59] Ruby P. Decety J. (2003). What you believe versus what you think they believe: a neuroimaging study of conceptual perspective-taking. *Eur. J. Neurosci.* 17 2475–2480. 10.1046/j.1460-9568.2003.02673.x 12814380

[B60] Salvatore G. Dimaggio G. Popolo R. Lysaker P. H. (2008). Deficits in mindreading in stressful contexts and their relationships to social withdrawal symptoms in schizophrenia. *Bull. Menninger Clin.* 72 189–207. 10.1521/bumc.2008.72.3.191 18990055

[B61] Scherzer P. Leveillé E. Achim A. Boisseau E. Stip E. (2012). A study of theory of mind in paranoid schizophrenia: a theory or many theories? *Front. Psychol.* 3:432 10.3389/fpsyg.2012.00432 PMC3497936 23162496

[B62] Searle J. R. (1975). Indirect speech acts. *Syntax Semant.* 3 59–82.

[B63] Shamay-Tsoory S. G. Shur S. Barcai-Goodman L. Medlovich S. Harari H. Levkovitz Y. (2007). Dissociation of cognitive from affective components of theory of mind in schizophrenia. *Psychiatry Res.* 149 11–23. 10.1016/j.psychres.2005.10.018 17107716

[B64] Shamay-Tsoory S. G. Tibi-Elhanany Y. Aharon-Peretz P. (2006). The ventromedial prefrontal cortex is involved in understanding affective but not cognitive theory of mind stories. *Soc. Neurosci.* 1 149–166. 10.1080/17470910600985589 18633784

[B65] Shamay-Tsoory S. G. Tomer R. Berger B. D. Goldsher D. Aharon-Peretz J. (2005). Impaired “affective theory of mind” is associated with right ventromedial prefrontal damage. *Cogn. Behav. Neurol.* 18 55–67. 10.1097/01.wnn.0000152228.90129.99 15761277

[B66] Stone V. Baron-Cohen S. Knight R. T. (1998). Frontal lobe contributions to theory of mind. *J. Cogn. Neurosci.* 10 640–656. 10.1162/089892998562942 9802997

[B67] Tager-Flusberg H. Sullivan K. (2000). A componential view of theory of mind: evidence from Williams syndrome. *Cognition* 76 59–89. 10.1016/S0010-0277(00)00069-X 10822043

[B68] Uhlhaas P. J. Phillips W. A. Schenkel L. S. Silverstein S. M. (2006). Theory of mind and perceptual context-processing in schizophrenia. *Cogn. Neuropsychiatr.* 11 416–436. 10.1080/13546800444000272 17354079

[B69] Wechsler D. (1997). *Wechsler Adult Intelligence Scale.* Third Edn San Antonio, TX: The Psychological Corporation.

[B70] Weimer A. A. Sallquist J. Bolnick R. R. (2012). Young children’s emotion comprehension and theory of mind understanding. *Early Educ. Dev.* 23 280–301. 10.1080/10409289.2010.517694

[B71] Wilson B. A. Alderman N. Burgess P. W. Emslie H. Evans J. J. (1996). *Behavioural Assessment of Dysexecutive Syndrome.* London: Harcourt Assessment.

[B72] Zalla T. Sav A.-M. Stopin A. Ahade S. Leboyer M. (2009). Faux pas detection and intentional action in Asperger syndrome. A replication on a French sample. *J. Autism Dev. Disord.* 39 373–382. 10.1007/s10803-008-0634-y 18726150

